# Development and evaluation of a desktop VR serious game for color composition learning in basic design education

**DOI:** 10.1371/journal.pone.0359618

**Published:** 2026-09-30

**Authors:** Jingru Sun, Jingru Zhang, Weichang Chen, Jingjing Fan, Fang Hao, Huasheng Lai

**Affiliations:** 1 School of Design and Art, Shandong Huayu University of Technology, Dezhou City, Shandong Province, China; 2 School of Design and Art, Jiangsu Ocean University, Lianyungang City, Jiangsu Province, China; 3 College of Fine Arts and Design, Quanzhou Normal University, Quanzhou City, Fujian Province, China; 4 College of Art and Design, Huainan Normal University, Huainan City, Anhui Province, China; 5 College of Landscape Architecture, Fujian Agriculture and Forestry University, Fuzhou, Fujian Province, China; University of Marburg: Philipps-Universitat Marburg, GERMANY

## Abstract

In recent years, immersive and interactive technologies have gained increasing attention in art and design education. Desktop virtual reality (VR) offers a low-cost and accessible alternative for classroom learning, yet its use in color composition education remains underexplored. This study aimed to develop and evaluate BD-VRG, a constructivist-based desktop VR serious game for color composition learning in basic design education. BD-VRG was developed using the ADDIE instructional design model and incorporated gamified interaction and real-time feedback within a low-cost desktop VR environment. A total of 42 first-year art and design students participated in the evaluation. Data were collected on learning performance, technology acceptance, usability, technical performance, post-exposure discomfort, and qualitative user feedback. A significant short-term improvement in post-test performance was observed (*d* = 0.82). All four Technology Acceptance Model dimensions were rated significantly above the neutral midpoint, and the System Usability Scale indicated good overall usability. The system also showed stable technical performance and low post-exposure discomfort. Qualitative feedback highlighted the value of real-time learning support and engaging practice, while also pointing to the need for clearer onboarding and navigation. Overall, the findings suggest that BD-VRG is a feasible and promising approach for supporting color composition learning in basic design education. These findings further indicate that low-cost, classroom-deployable desktop VR serious games may support meaningful perceptual and interactive learning for abstract design concepts, thereby offering an accessible and pedagogically valuable approach to art and design education.

## Introduction

Basic design courses play a foundational role in art and design education, serving to enhance students’ approaches to learning and their cognitive abilities regarding fundamental design principles [1]. This encompasses the comprehension of design elements like shape, color, texture, light, and rhythm [2]. Among these, color harmony is a core concept that underpins a wide range of design practices, including graphic design, product design, and visual communication. In addition to acquiring theoretical knowledge, the purpose is to foster an aesthetic sense and appreciation of beauty, enabling learners to develop creative thinking and the ability to produce original works [3]. Although this course is crucial to design education, students often lack interest in theoretical knowledge, making it difficult for them to grasp abstract concepts such as tonal relationships, contrast, harmony, and emotional impact [4]. Traditional teaching methods frequently rely on static tools with limited interactivity, which may hinder students’ intuitive understanding of color interactions and the application of theoretical knowledge in design practice.

Against this background, there remains a need for tools that can inspire students to actively engage in their education and generate their own knowledge rather than merely absorb it. At present, the continuous evolution of digital and immersive technologies is profoundly reshaping the landscape of educational technology [5]. In education, virtual reality (VR) is increasingly used to develop experiences [6]. Such immersion significantly enhances the learning process by allowing students to interact with knowledge in more meaningful and memorable ways [7]. In addition, serious games (SGs), which employ enjoyable experiences to assist students in reaching their learning objectives, are gaining popularity [8]. VR-based serious games, by incorporating immersion, interactivity, and feedback systems, aim to overcome the constraints of two-dimensional teaching materials and conventional classrooms, thereby allowing students to experiment and explore at their own pace. Through gamification mechanisms, such as scoring, challenges, and levels, these systems offer an alternative approach to facilitating the understanding of abstract design concepts and enhancing learner engagement [9]. However, most VR-based design education systems rely on head-mounted displays (HMDs), limiting classroom adoption, whereas desktop VR enables immersive interaction without HMDs and offers greater accessibility through low cost and easy deployment, leading to its increasing use in classroom instruction [10]. However, empirical evidence remains limited regarding how desktop VR–based serious games can be systematically designed and evaluated for abstract learning tasks such as color composition learning in basic design education.

The effective integration of VR-based serious games into formal education requires careful consideration of pedagogical alignment and collaboration between educators and system developers [11]. Moreover, the development of such learning environments depends on user-friendly interface design and an appropriate balance between open-ended artistic exploration and structured learning tasks. Accordingly, systematic evaluation mechanisms that integrate usability, acceptance, and learning-related indicators are essential to assess their educational value and support data-driven improvements in user experience and instructional effectiveness [12,13].

To address these challenges, this study developed a desktop VR serious game learning system, BD-VRG, for color composition learning. Grounded in constructivist theory, the system integrates interactive game tasks, immersive exploration, and instant visual feedback, aiming to enhance students’ understanding of color composition by providing a more engaging, intuitive, and student-centered learning environment. Unlike prior studies that primarily rely on head-mounted displays or focus on single-dimensional evaluations, this study adopts a multi-dimensional evaluation approach with a particular focus on usability and acceptance, alongside an exploratory examination of learning-related outcomes.

Accordingly, this study aimed to (1) design and implement a low-cost and accessible desktop VR serious game for color composition learning in basic design education; (2) examine the system’s usability and students’ technology acceptance using established evaluation instruments; and (3) assess short-term learning gains in color composition knowledge immediately following system use, as reflected in pre-/post-test performance. In addition, prior gaming experience was examined as a contextual factor in the analysis.

## Literature review

### Digital technology in basic design education

The basic design course originated from the “Vorkurs” (preparatory foundation course) established by Walter Gropius at the Bauhaus School in Germany in 1919, which is a crucial starting point in art and design education [14]. By improving students’ learning approaches and cognitive capacities related to fundamental design concepts, basic design education enhances their comprehension and creativity and equips them to produce effective designs across a variety of fields [15]. Digital technologies have increasingly been introduced into basic design education to support more interactive and visually enriched learning experiences [16]. Özgen et al. [17] found that the VR group demonstrated significantly higher “intention to use” and “perceived enjoyment” in basic design tasks than the paper-based group. The VR group also exhibited greater effectiveness in problem-solving tasks, indicating that VR provides significant advantages as a supplementary tool in basic design education. Studies have also shown that immersive technologies such as AR and MR can improve students’ learning attitudes, intention to use, perceived enjoyment, and exploratory abilities in basic design education [18,19]. In addition, AI is emerging as a valuable tool in basic design education, and these applications can complement traditional form-making exercises by enabling rapid iteration and providing creative inspiration [20].

Overall, digital technology has profoundly transformed teaching methods and learning experiences in basic design education [21]. Recent developments have increasingly emphasized the integration of interactive and immersive digital technologies to support visualization, engagement with authentic design tasks, and creative exploration [22]. However, existing studies largely emphasize technological affordances or general learning attitudes, while limited attention has been paid to the systematic design and evaluation of immersive digital systems for abstract learning tasks such as color composition learning, particularly in accessible, classroom-friendly formats. Consequently, perceptual dimensions of color harmony, including relational contrast, balance, and emotional resonance, remain difficult to grasp through conventional digital tools. This gap highlights the need for interactive and experiential learning environments that provide visual support and pedagogical structure for foundational color composition learning.

### VR-based serious games in education

A serious game is a game in which the primary objective is educational rather than purely entertainment-oriented [23]. In recent years, VR-based serious games have attracted growing attention as instructional tools because they balance entertainment with educational objectives, combine immersion, interactivity, and challenges to facilitate learning while maintaining enjoyment [24,25]. Prior studies across fields such as medical chemistry and science education have shown that VR serious games can enhance learner engagement, support interactive exploration, and promote immediate understanding of complex content [24,26,27]. In art and design, researchers have explored the use of VR serious games in cultural heritage learning, with review evidence highlighting gamification and immersive exploration in virtual museums and architectural site visits [8, 28]. Another study introduced the Design Review Simulator (DRS), a VR serious game based on the ADDIE framework, to support architectural design review of residential buildings [29]. Collectively, these examples highlight the broad educational potential of VR-based serious games across diverse disciplines; however, their classroom implementation is influenced by the affordances and practical constraints associated with different technological configurations.

VR-based serious games employ diverse technological configurations, ranging from fully immersive head-mounted display (HMD)-based VR systems to semi-immersive or desktop VR environments, which may result in substantially different learning experiences and instructional affordances [30]. Although HMD-based VR is often valued for its high level of immersion and spatial presence, previous studies have also identified practical and pedagogical limitations, including high hardware costs, limited accessibility, motion sickness, and increased cognitive load during prolonged use [31,32]. Given that color composition learning requires fine-grained visual perception, judgments of color harmony, and repeated manipulation of visual elements [33], the selection of VR configurations should account for display constraints, visual fatigue, and attentional demands. Previous studies have applied desktop VR in art and design education primarily to 3D modeling, art appreciation, virtual exhibitions, and spatial visualization [34–36], while many have focused on functional visualization or instructional demonstration, with limited integration of game-based instructional structures such as explicit learning objectives, challenges, and assessment mechanisms. Empirical research on desktop VR serious games for color composition learning remains scarce. Taken together, these considerations indicate the need for a systematic comparison among HMD-based VR serious games, desktop VR-based serious games, and traditional instruction to clarify their relative suitability for color composition education (Table 1).

**Table 1:** Conceptual comparison of learning approaches.

Dimension	HMD-based VR serious game	Desktop VR–based serious game	Traditional instruction
Cost	Higher; requires specialized HMD equipment [34]	Lower; uses conventional desktop computers and input devices [35,36]	Lowest; requires no VR-specific hardware [17]
Accessibility	Lower; limited device availability; potential cybersickness [31,32]	Higher; requires no wearable equipment and is easier to implement in educational settings [34]	High; broadly accessible through conventional classroom materials [17]
Level of abstraction	Lower; provides highly immersive visualization of complex concepts [17,30]	Moderate; provides interactive 3D visualization through a conventional screen [10,34]	Higher; abstract theoretical concepts may remain less visually concrete [17,34]
Cognitive load	Higher; immersive visual input and device operation may increase cognitive demands [30,31]	Moderate; familiar controls may reduce task effort relative to non-ambulatory HMD VR [10]	Lower; technology-related demands, although abstract theoretical concepts may remain cognitively demanding [4,17]

Table 1 presents a conceptual comparison across key dimensions, including cost, accessibility, level of abstraction, and cognitive load. While HMD-based VR offers high immersion, its high cost, limited accessibility, and potential cognitive overload may limit its suitability for foundational color learning in classroom contexts. In contrast, desktop VR enables immersive 3D interaction without specialized immersive hardware and supports abstract color concepts through controllable visualization. Compared with traditional instruction, desktop VR-based serious games offer greater visual concreteness and interactivity, which may support perceptual tasks such as color harmony learning. Taken together, these considerations support the present study’s focus on desktop VR as a low-cost and readily deployable means of integrating immersive and interactive learning into basic design classrooms.

### Color composition learning from a constructivist perspective

Constructivist learning theory posits that knowledge is actively constructed by learners through interaction with tasks, materials, and contextual environments, rather than passively received from instruction alone [37]. In this view, learning is a process of meaning-making in which learners integrate new information with prior knowledge through exploration, reflection, and problem-solving in authentic or semi-authentic contexts [38]. Constructivism has been widely applied in art and design education, where project-based, research-based, and collaborative learning approaches enable students to construct meaning through making, reflection, and dialogue rather than by copying models [39].

Color composition learning in basic design education aligns closely with constructivist assumptions. Concepts such as color harmony, contrast, balance, and emotional resonance are inherently relational and perceptual, and cannot be fully understood through rule-based instruction or static visual representations alone [40]. Instead, learners develop an understanding of color relationships through iterative experimentation, goal-directed practice, and contextual learning, gradually constructing internal cognitive frameworks that connect perceptual experience with conceptual knowledge [41]. Moreover, constructivist theory highlights the importance of interaction and feedback in supporting conceptual development [42]. In the context of color learning, immediate visual feedback and opportunities for manipulation allow learners to test assumptions, observe perceptual effects, and refine their understanding of color relationships through trial and error. Such interactive processes are particularly relevant to design tasks involving abstract concepts and perceptual judgments, for which experiential exploration can complement explicit instruction [41]. Accordingly, applying constructivism to color composition learning requires not only providing environments that support exploration, task-driven interaction, and reflective engagement but also aligning these activities with explicit learning objectives to facilitate meaningful learning [39,41].

### Technology Acceptance Model (TAM)

The Technology Acceptance Model (TAM) is a widely used and theoretically grounded framework for explaining users’ acceptance of digital technologies [43,44]. It comprises four key constructs: perceived usefulness (PU), perceived ease of use (PEOU), attitude toward using (ATT), and behavioral intention to use (BI). According to TAM, users’ acceptance of a system is shaped by their perceptions of its usefulness and ease of use, which in turn influence attitude and future intention to use the technology [43,45]. PU refers to the extent to which users believe that a system enhances their learning performance, whereas PEOU reflects the degree to which the system is perceived as easy to use. Moreover, TAM posits that PEOU serves as a predictor of PU, and that users’ attitudes (ATT) toward a system also influence their intention to use the technology.

TAM has been widely applied in studies of educational technologies and digital games, including mobile VR and other game-based learning systems [46]. In a design-related context, Wang et al. [4] employed TAM to evaluate a mobile color-mixing game and reported positive learner perceptions across all four TAM dimensions, accompanied by increased engagement and cognitive outcomes. More recent research involving art students similarly identified perceived usefulness as a positive predictor of continuance intention [47]. Accordingly, TAM was adopted in the present study to evaluate learners’ acceptance of the proposed desktop VR serious game for color composition learning.

## Methods

### Participants

A total of 42 first-year art and design students, aged 18–21 years, were recruited for the usability evaluation. All participants were enrolled in a required basic design course. The researcher inquired about participants’ experience with VR games or similar activities to confirm their computer skills. Twenty-six participants had VR gaming experience, while the remaining participants did not. None of the participants had prior exposure to any VR serious gaming instructional system. Because 62% of the participants reported prior gaming experience, subgroup analyses were subsequently conducted to examine whether this background characteristic was associated with differences in the study outcomes.

Ethical approval for this study was granted by the Ethics Review Committee of Jiangsu Ocean University (Reference No. 2025-HUM-093001) prior to participant recruitment. Participants were recruited from 11 October 2025–21 October 2025. All participants were adults and provided written informed consent before taking part in the study. Participation was entirely voluntary, and participants were informed that they could withdraw at any time without penalty and that non-participation would not affect their course grades or academic standing. No personally identifying information was collected, and all data were analyzed anonymously and stored securely for research purposes only.

To examine whether the sample size was adequate for detecting the observed pre–post difference in learning performance, a post hoc power analysis was conducted using G*Power. For the paired-samples t test, with an observed effect size of Cohen’s *d* = 0.82, an alpha level of .05, and a total sample size of 42, the achieved power was greater than .99, indicating that the present sample size was sufficient to detect the observed effect.

### Development of the BD-VRG System

The BD-VRG system was developed using the ADDIE instructional design model, which guided the analysis, design, development, implementation, and evaluation of the system (Fig 1).

**Fig 1:**
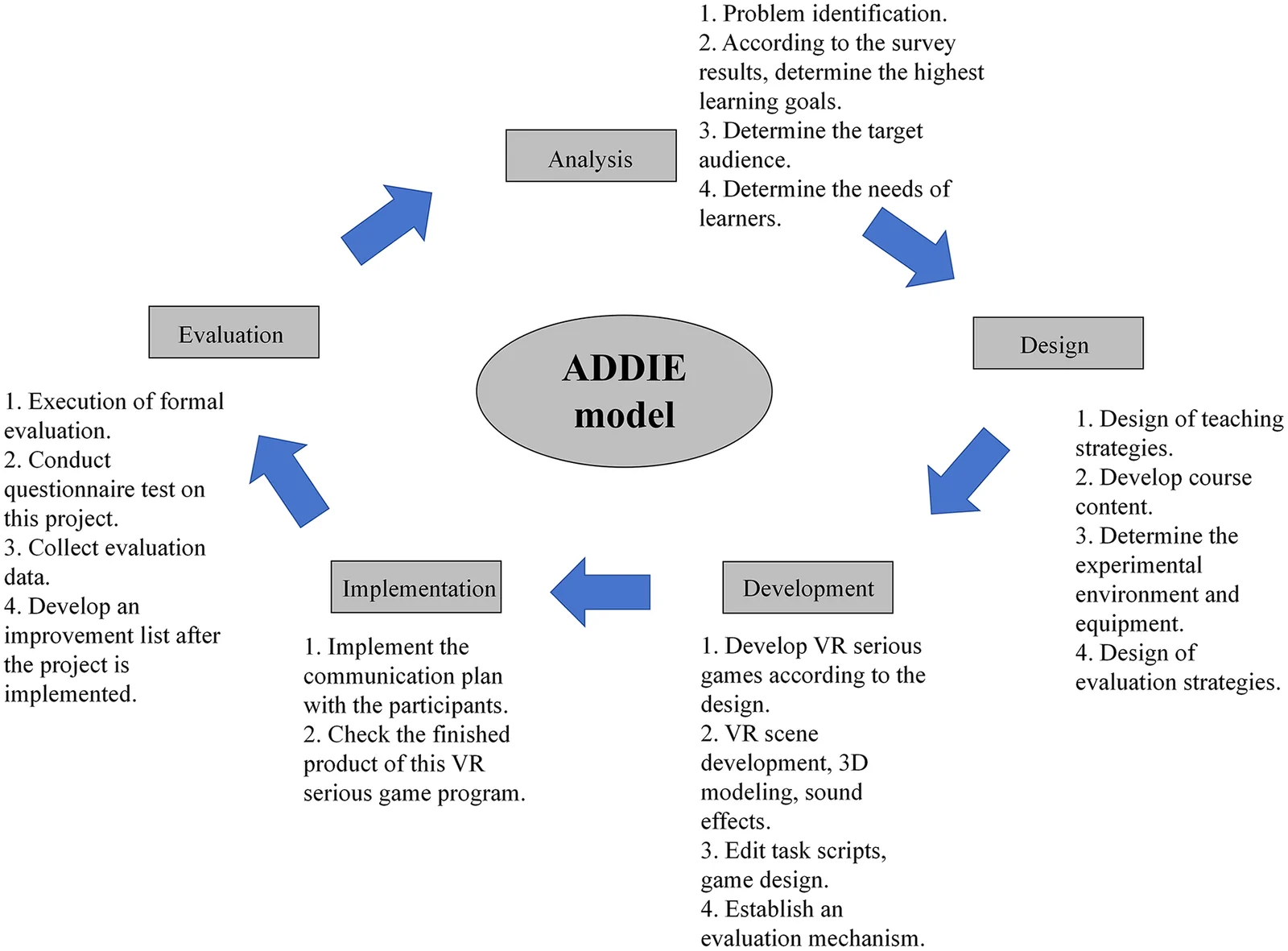
BD-VRG system development flowchart.

#### Analysis.

The analysis phase aimed to identify the instructional objectives and learner needs for a desktop VR serious game for color composition learning. After the literature review, a needs assessment was conducted using semi-structured interviews with two lecturers of a basic design course and open-ended questionnaires completed by 25 first-year art and design students from the target user group. The responses were thematically analyzed using an inductive coding approach to identify recurring needs and learning barriers related to color composition learning. The main problems identified were that color theory is abstract and difficult to understand; traditional teaching methods lack interactivity and rely on static tools for formal exercises; and students lack motivation to learn. The resulting raw themes and their frequencies are reported in Appendix A (S1 File) to ensure traceability. Finally, based on the needs assessment findings, the characteristics of the target learning group were analyzed (Fig 2).

**Fig 2:**
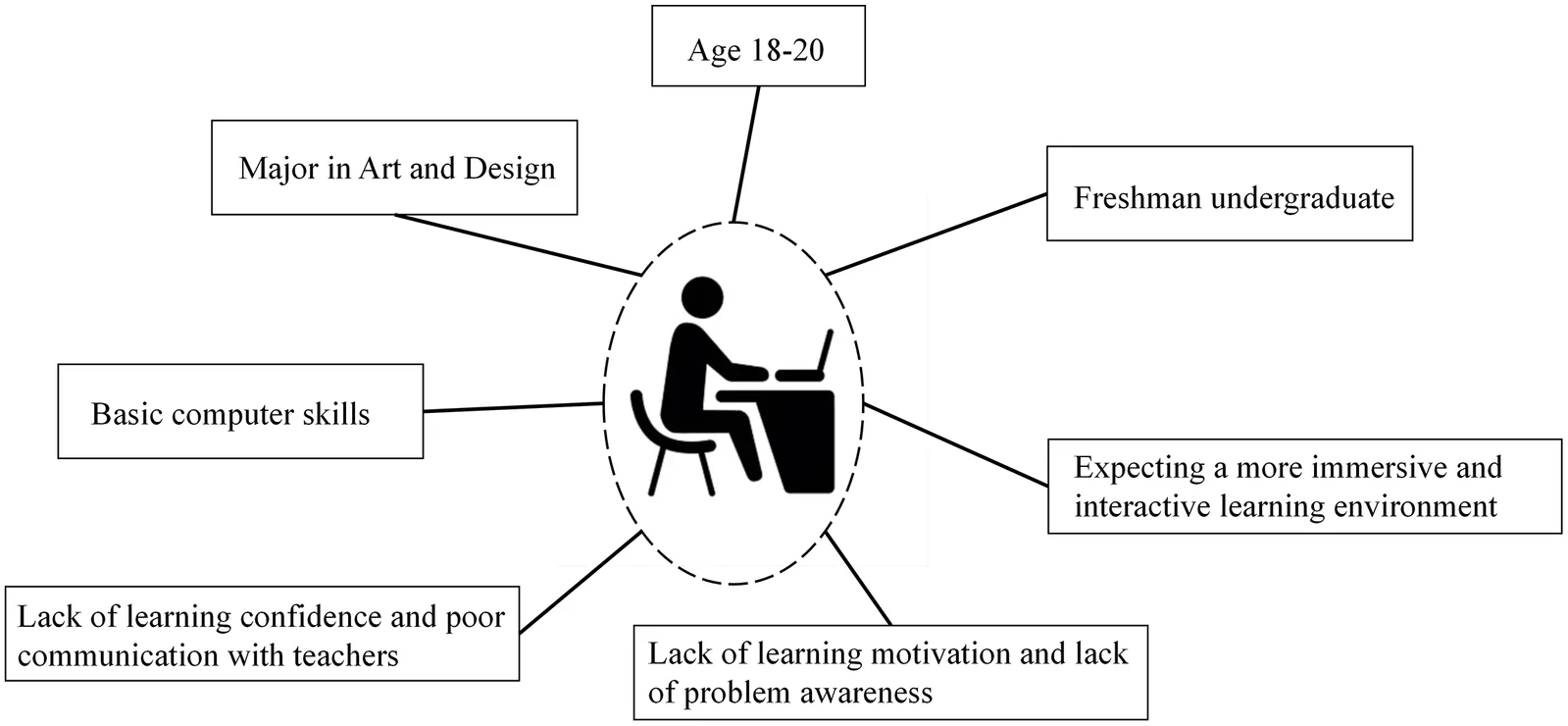
Learner analysis.

The target learning group consists of first-year art and design students with basic computer-operation skills and seeks a more immersive and interactive learning experience. Accordingly, the instructional objectives of BD-VRG were defined as follows: to help students master basic color concepts (such as hue, purity, and brightness); engage in interactive color practice in a virtual environment; and improve students’ ability to apply and express color composition rules through tasks embedded in serious games.

#### Design.

Based on the analysis results, the BD-VRG system was designed as a desktop VR serious game for color composition learning in basic design education. Guided by constructivist learning theory, the system emphasized task-oriented exploration, gamified practice, immediate feedback, and a reward system within an interactive virtual environment. Design decisions were informed by the extracted requirements (Appendix A in S1 File), and the requirement-to-design mapping is summarized in Table A in S1 File to ensure traceability.

The course content was developed based on information gathered from the interviews and an analysis of textbook content. The BD-VRG system focused on three core themes: foundational knowledge of color, emotional expression of color, and visual perception of color composition. These themes were selected as representative components of color composition learning in basic design and were considered suitable for integration with gameplay. Across modules, learning followed an explain-practice-feedback-application sequence, in which students first learned core concepts and then completed corresponding game-based tasks to reinforce understanding. In the foundational module, students learned basic concepts such as hue, brightness, and purity and then completed related tasks such as sorting and synthesis. In the color emotion module, students constructed emotional keywords based on color themes and explored how color combinations conveyed particular emotions or thematic meanings. In the visual perception module, students practiced judging overall coordination, rhythm, and color matching in composition. A reward system, including scores, badges, repeatable challenges, and progress tracking, was incorporated to support motivation, sustained engagement, and the application of abstract color theory concepts in practice.

To support classroom deployment, the system was designed as a desktop VR application that operated without a head-mounted display. The study was conducted in a university computer laboratory using standard desktop computers, 24-inch full HD monitors, keyboard-and-mouse input, and headphones with an emphasis on screen interaction and depth perception. Stereo audio was delivered through headphones to enrich immersive sound effects and instructional narration. The researchers were present to provide essential technical support. This desktop VR setup was selected to satisfy the low-barrier classroom deployment requirement (Appendix A in S1 File) by delivering 3D immersive learning experiences via standard computer interfaces, thereby helping to reduce adoption barriers and enable classroom integration.

#### Development.

The BD-VRG system was developed in Unity 3D, integrating instructional content, game-based tasks, and 3D model design, with C# scripts used to implement the interactive logic. BD-VRG did not employ a predictive, optimization, or adaptive mathematical model; instead, learner actions and task flow were managed through predefined interaction rules, as illustrated in Fig 3.

**Fig 3:**
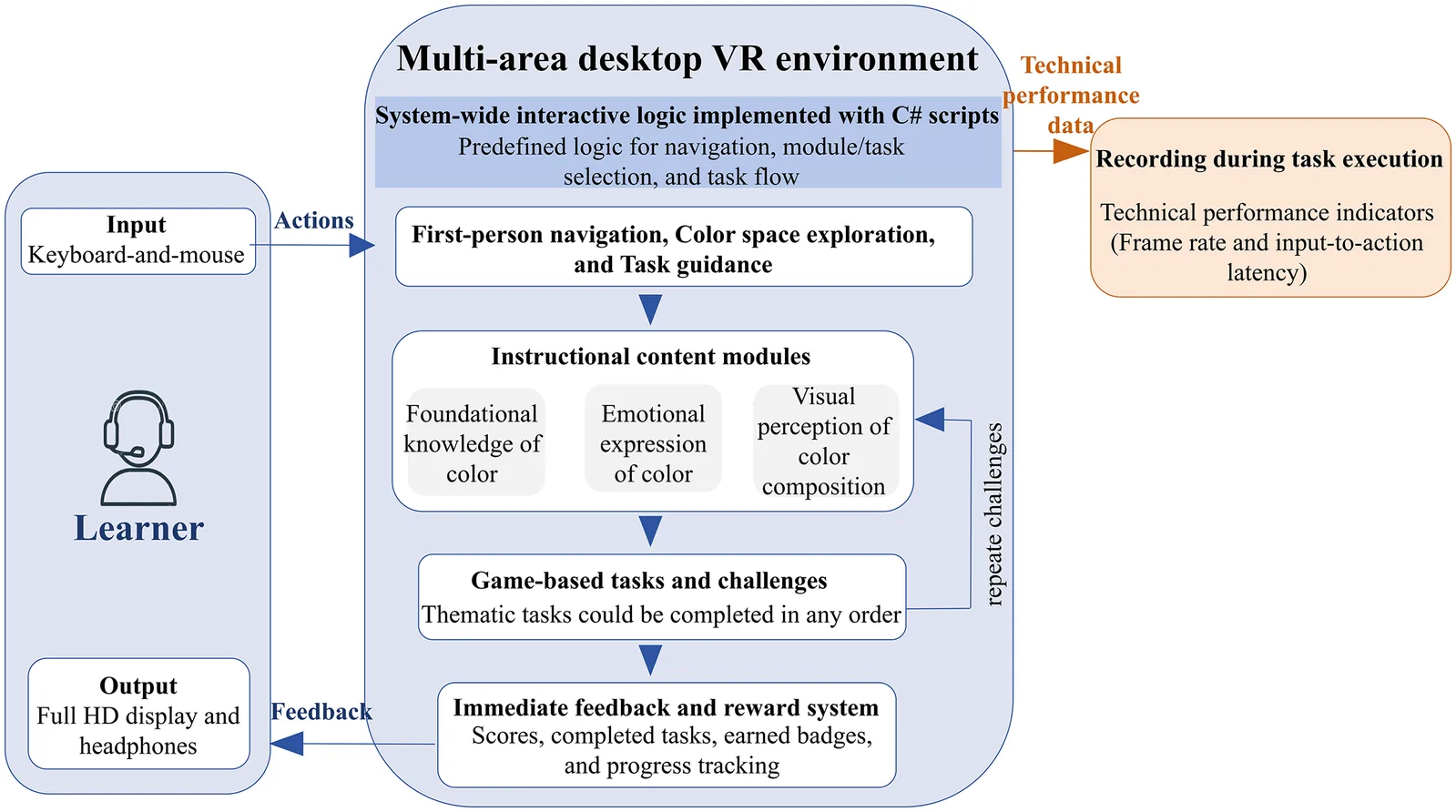
Architecture and instructional flow of the BD-VRG system.

To support immersive and user-friendly color composition learning, a multi-area desktop VR environment was constructed. Interactive elements, including the color materials wall and the experimental table, were created as 3D models based on actual color experimentation instruments. Students used a first-person mode to navigate the environment, beginning with the theoretical module to learn core concepts related to the color wheel, emotional expression, and visual perception through an interactive display. After completing the theoretical content, they proceeded to corresponding game-based tasks and received immediate feedback and scores. The system displayed total score, completed tasks, and earned badges to support motivation, and students could complete thematic tasks in any order or repeat challenges to improve their performance. To prevent unintentional exits or shutdowns, a pop-up window was used to provide confirmation prompts. Visual guidance cues were provided throughout the learning process to support intuitive navigation and task completion (Fig 4).

**Fig 4:**
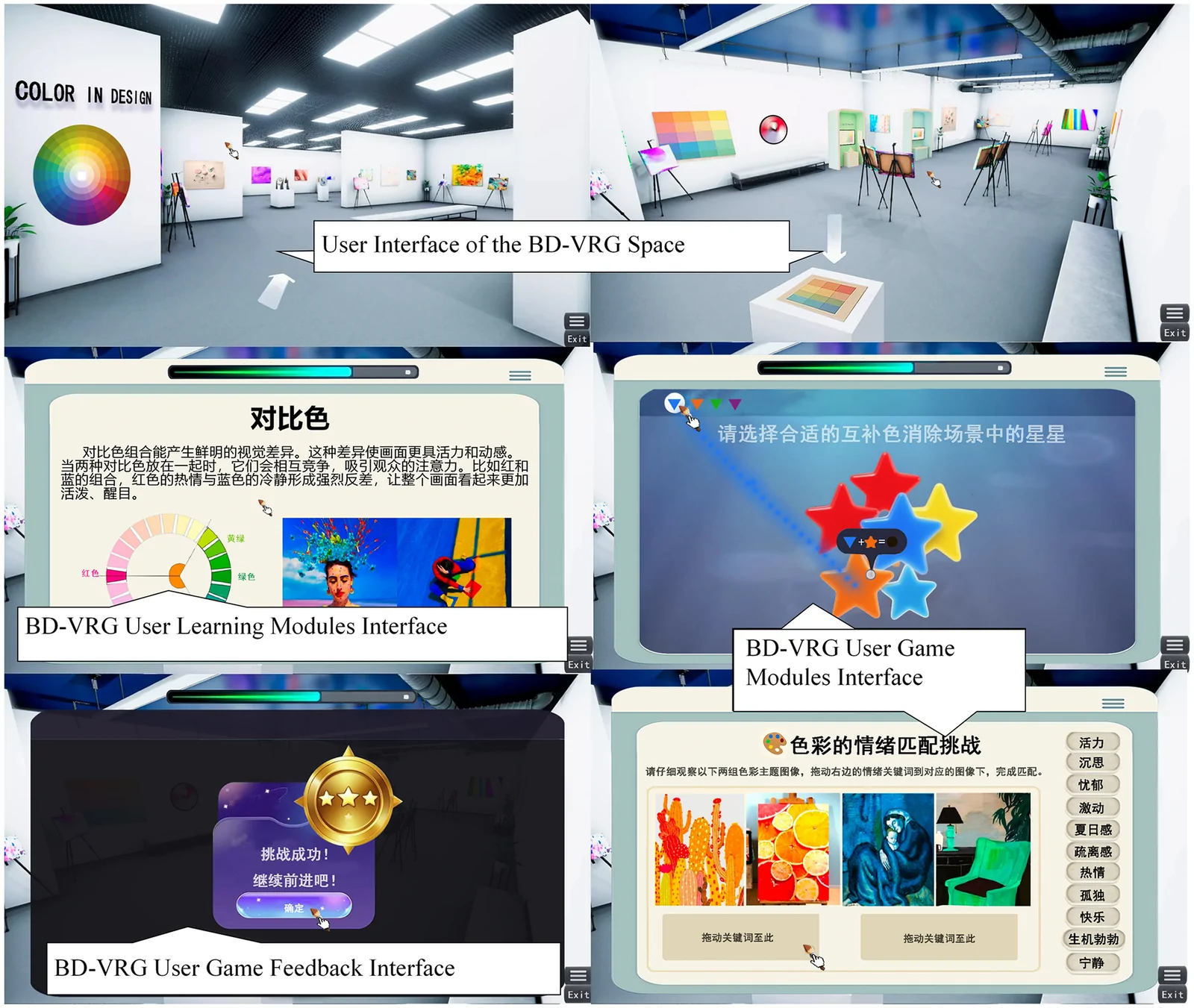
BD-VRG system user interface design.

The prototype supported key actions, including color selection, color modification, color composition, task feedback, timed challenges, and scoring. Interface layouts were kept consistent across modules to support navigation and usability. The instructional content was developed in consultation with educational experts to improve its pedagogical relevance. To support subsequent technical evaluation, the prototype was instrumented to record technical performance indicators, including frame rate and input-to-action latency, during task execution. The finalized system was packaged as a standalone Windows executable for use in the experimental study. Additional details of the iteration process are reported in Appendix C (S1 File).

#### Expert review and refinement.

Before formal evaluation, the BD-VRG design draft, including the learning objectives, module structure, task flow, feedback and reward logic, and desktop VR configuration, was reviewed by two domain experts in two rounds. The expert panel consisted of a postdoctoral researcher in digital media art and a user interface design director, providing complementary expertise in pedagogy, content, and interface design. Both experts had more than six years of relevant experience.

The two-round review followed a formative and confirmatory process. The first round identified revision priorities related to instructional alignment, interaction design, feedback clarity, and classroom feasibility. After revisions were implemented, the second round reassessed the revised design using the same checklist to confirm the adequacy of the updates and readiness for subsequent development. Inter-rater agreement on the experts’ decision labels (A/R/X) exceeded 0.80 in both rounds (Round 1 κ = 0.86; Round 2 κ = 0.83; Appendix B in S1 File). The major revisions and their corresponding design updates are summarized in Table B in S1 File. Their feedback informed revisions to instructional flow, terminology consistency, feedback clarity, reward logic, error prevention, and difficulty progression, and the revised prototype was then finalized for subsequent evaluation.

### Procedure

A preliminary trial was conducted after development to verify the system’s basic operability and the effectiveness of its teaching logic. The study was conducted in a controlled university computer laboratory. Each participant independently completed the full experimental session, which took approximately 80 minutes per individual (Fig 5). Participants were welcomed and briefly informed about the study’s purpose. After the initial briefing, participants completed the CCKT pre-test (15 minutes) to assess their baseline color composition knowledge. Basic operating instructions were then provided for 5 minutes to help participants familiarize themselves with the navigation, task flow, and game rules of the BD-VRG system. No instructional content was delivered at this stage, and no assistance was provided during the game to maintain ecological validity.

**Fig 5:**
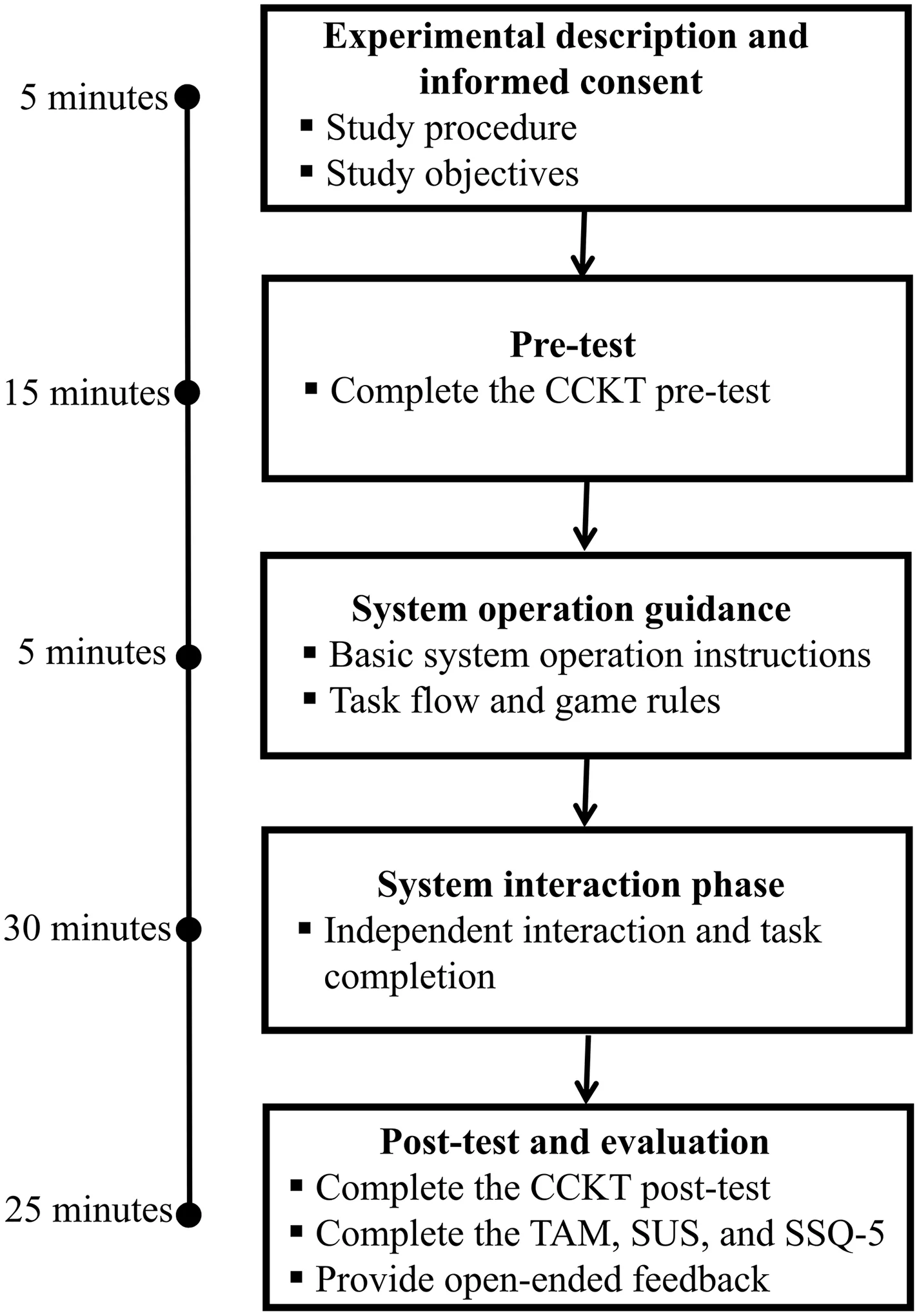
Experimental flowchart.

Subsequently, participants independently interacted with the BD-VRG learning system using a desktop-based VR setup. They explored the learning environment, completed the game tasks for each module, and monitored their progress using the system’s integrated feedback and scoring interface. The interactive portion was self-directed and lasted approximately 30 minutes. The researcher provided assistance if participants encountered difficulties using the equipment.

After completing the system interaction phase, participants completed the CCKT post-test and post-interaction evaluation (25 minutes). During this stage, participants completed the TAM, SUS, and SSQ-5 questionnaires, and also responded to four open-ended questions concerning learning support, unmet needs, operational difficulties, and interface improvement. The researcher then addressed any questions from the participants and expressed gratitude for their involvement.

### Instruments

#### Learning performance test.

The Color Composition Knowledge Test (CCKT) was a researcher-developed instrument designed to assess participants’ short-term gains in core color composition knowledge following interaction with the BD-VRG system. It was administered immediately before and after the experimental session.

Two parallel forms were developed for the pre-test and post-test. Each form consisted of 20 multiple-choice items covering the same core domains of color composition knowledge, including color matching, complementary and analogous color relationships, warm–cool contrast, emotional associations of colors, and judgments of overall harmony. The CCKT was developed from a test blueprint based on the course learning objectives, the instructional content of the BD-VRG modules, and established color-composition teaching materials. Matched item pairs assessed the same target concepts while varying the wording, design context, and response options to reduce potential item-recall effects. Each item required participants to select the most appropriate color combination or compositional relationship from four response options. One point was awarded for each correct answer, resulting in a total score ranging from 0 to 20. Raw scores were converted into accuracy percentages, and each form required approximately 15 minutes to complete.

To establish content validity, both forms were reviewed by two art and design experts with more than 10 years of teaching experience. The experts confirmed the relevance, clarity, and alignment of the items with the intended learning objectives, as well as the comparability of the two forms in content coverage and intended difficulty. Following expert review, the items underwent a small-scale cognitive pretest with six first-year art and design students who did not participate in the formal study. The students completed both forms, commented on wording clarity and response-option ambiguity, and their completion time was recorded. The pretest focused on comprehensibility and administration feasibility rather than psychometric evaluation. Based on their feedback, the wording of Pre-test Item 3 was revised from “Which colors show warm and cool differences?” to “Which color combination would most clearly express a warm–cool contrast in a composition?” to clarify the required judgment. In Post-test Item 5, the distractor “Combining warm and cool colors with controlled saturation and balanced proportions” was identified as potentially harmonious and was replaced with “Mixing warm and cool colors randomly to increase complexity,” thereby reducing conceptual overlap with the correct answer.

After formal administration, item-level descriptive analyses were conducted separately for the pre-test and post-test forms using the present study data. Item difficulty was defined as the proportion of participants answering each item correctly, and item discrimination was examined using corrected item–total correlations. Both forms showed a moderate range of item difficulty, and all corrected item–total correlations were positive (Table C in S1 File). Given the dichotomous scoring format, internal consistency was estimated separately using the Kuder–Richardson Formula 20 (KR-20). The KR-20 coefficients were 0.72 (95% CI [0.57, 0.83]) for the pre-test form and 0.75 (95% CI [0.63, 0.85]) for the post-test form. Because these estimates were based on a modest sample of 42 participants, they should be interpreted as preliminary. Representative paired items and the test blueprint are provided in Appendix D (S1 File).

#### Technology Acceptance Model (TAM) questionnaire.

In this study, the TAM questionnaire was adapted from the original tool developed by Davis et al. [43] and from questionnaires used in previous studies related to serious games [4]. The instrument consists of 15 items and evaluates users’ acceptance of the BD-VRG system across four dimensions: PU (4 items), PEOU (4 items), ATT (4 items), and BI (3 items). The original items were modified to fit the context of VR-based serious games for teaching color composition in basic design education. All items in the modified TAM questionnaire were rated on a 7-point Likert scale, with 1 indicating strongly disagree and 7 indicating strongly agree. Each participant’s score for a dimension was calculated by taking the mean of all item ratings within that dimension. Higher scores indicate greater approval of the system regarding usefulness, ease of use, positive attitude, and intention to use.

To assess the internal consistency of the adapted TAM questionnaire, Cronbach’s alpha coefficients were calculated for the four dimensions. The alpha values were 0.74 for PU (95% CI [0.58, 0.85]), 0.78 for PEOU (95% CI [0.64, 0.87]), 0.82 for ATT (95% CI [0.71, 0.89]), and 0.80 for BI (95% CI [0.66, 0.89]), indicating acceptable internal consistency across the four dimensions. The questionnaire also included two open-ended questions to allow participants to provide suggestions and comments after the VR game test. Before data collection, two specialists in art and design and educational technology, each with over five years of experience in their respective fields, reviewed the questionnaire and confirmed that the instrument was appropriate for this study. To further examine the structural properties of the adapted TAM questionnaire, an exploratory factor analysis (EFA) was conducted on the 15 items using principal axis factoring with Promax rotation.

#### System Usability Scale (SUS).

The System Usability Scale (SUS) [48] was used to assess the overall usability of the BD-VRG system. A study reviewing ten years of SUS score data found that it is a highly robust and adaptable instrument for usability experts [49]. The SUS consists of 10 items with alternating positive and negative statements, rated on a 5-point Likert scale from 1 (strongly disagree) to 5 (strongly agree). Following the standard SUS scoring procedure, for positively worded items (Items 1, 3, 5, 7, 9), the score contribution is calculated by subtracting 1 from the user response. For negatively worded items (Items 2, 4, 6, 8, 10), the score contribution is calculated by subtracting the user response from 5. All item scores were summed, then multiplied by 2.5 to obtain a total score ranging from 0 to 100, with higher scores indicating better perceived usability. In the present study, the mean, standard deviation, and 95% confidence interval of the SUS total score were reported, and the result was interpreted with reference to the commonly used benchmark score of 68. Two open-ended questions were also included to collect participants’ feedback on operational difficulties and interface improvement needs; these responses were analyzed separately in the qualitative findings section.

#### System technical performance and post-exposure discomfort assessment.

To assess whether usability and acceptance outcomes might have been affected by technical instability, system-level performance indicators were recorded during testing. Specifically, average frame rate (FPS) and input-to-action latency were measured in the finalized BD-VRG prototype. FPS was recorded across 15 independent 30-minute continuous runs to evaluate rendering stability during prolonged operation. Input-to-action latency was defined as the elapsed time between a participant’s mouse click and the corresponding system response.

To screen for post-exposure discomfort following short-duration desktop VR use, a five-item checklist was administered immediately after the experiment (SSQ-5). The checklist was based on selected items from the Simulator Sickness Questionnaire (SSQ), a widely used 16-item instrument for assessing simulator-related discomfort, including symptoms associated with nausea, oculomotor strain, and disorientation [50]. This abbreviated checklist was used in the present classroom-based study to reduce participant burden. Responses were recorded on a 5-point Likert scale, and the overall score was calculated as the mean of the five items, with higher scores indicating greater self-reported post-exposure discomfort. The selected items were reviewed by two experts in VR-based learning and interface design to ensure their relevance and clarity for this study context. The five-item checklist showed acceptable internal consistency in the present sample (Cronbach’s α = 0.75, 95% CI [0.60, 0.85]). However, this abbreviated checklist was used for screening purposes only and was not intended to replace the full SSQ, reproduce its subscale structure, or support diagnostic interpretation. Accordingly, the results were analyzed descriptively and interpreted alongside participant completion status.

### Data analysis

Statistical analyses included paired-samples, independent-samples, and one-sample t tests, and Mann–Whitney U tests. Reliability was assessed using Cronbach’s alpha and KR-20. Exploratory factor analysis and bootstrap path analysis were conducted for the adapted TAM questionnaire. Technical performance indicators and post-exposure discomfort scores were summarized descriptively. All quantitative analyses were performed using SPSS 27 and AMOS 31. These packages were selected because they are widely used in educational and behavioral research, offer well-established and complementary analytical capabilities, and support the prespecified analyses in this study. Statistical significance was set at *p* < .05.

To complement the quantitative findings, open-ended responses collected at the end of the TAM and SUS questionnaires were analyzed thematically following Braun and Clarke’s [51] six-phase approach. The four open-ended questions asked participants about the system’s support for color design learning, unmet learning needs, operational difficulties, and suggestions for interface or operational improvement. All responses were imported into NVivo 14 for coding and organization (*N* = 42). Two independent coders analyzed the data through an iterative process. All responses were read repeatedly to achieve familiarity with the dataset, after which meaningful units were assigned initial codes and similar codes were grouped into broader candidate themes. The coders then reviewed and refined the developing themes by comparing them against the coded extracts and the full dataset, and the final themes were defined and named through discussion until consensus was reached. Inter-coder reliability was assessed using Cohen’s kappa, yielding κ = 0.78, indicating good agreement. Representative quotations were retained to enhance the transparency of the analysis and to illustrate the meaning of each theme. Theme frequency was calculated as the number of participants who mentioned a given theme at least once. Because individual participants could mention more than one theme, the total percentage exceeded 100%.

## Results

A total of 42 participants completed the evaluation of the BD-VRG system. The results are presented in two parts. First, the quantitative findings are reported, including learning performance, subgroup comparisons, technical performance, technology acceptance, and usability. This is followed by the qualitative findings derived from participants’ open-ended responses, which are presented to supplement and contextualize the quantitative results. Together, these findings provide a comprehensive understanding of the system’s learning support, usability, user acceptance, and areas for further refinement.

### Learning performance results

To examine whether participants demonstrated measurable improvement in learning performance after using the BD-VRG system, pre- and post-test scores on the Color Composition Knowledge Test (CCKT) were compared. Prior to the inferential analysis, the normality of the gain scores (post-test minus pre-test) was assessed using the Shapiro–Wilk test. The result indicated that the gain scores did not significantly deviate from normality (*W* = .960, *p* = .148), supporting the use of a paired-samples t test. The results are presented in Table 2.

**Table 2:** Pre-test and post-test results for the Color Composition Knowledge Test.

Measure	Pre-test	Post-test	Mean difference (95% CI)	*t* (df)	*p*	Cohen’s *d*
CCKT Accuracy (%)	50.12 ± 19.71	61.67 ± 20.41	11.55 [7.16, 15.93]	5.32 (41)	< .001	0.82

As shown in Table 2, participants’ CCKT accuracy improved from 50.12% (*SD* = 19.71) at pre-test to 61.67% (*SD* = 20.41) at post-test. The mean gain was 11.55 percentage points. A paired-samples *t* test revealed that this improvement was statistically significant, *t*(41) = 5.32, *p* < .001, 95% CI [7.16, 15.93], Cohen’s *d* = 0.82. These findings indicate that interaction with the BD-VRG system was associated with a significant short-term improvement in participants’ color composition learning performance.

### Subgroup analysis by prior gaming experience

Given that 62% of the participants reported prior gaming experience, subgroup analyses were conducted to examine whether this background characteristic influenced the study outcomes. An independent-samples *t* test was conducted to compare learning gain between participants with and without prior gaming experience, and Mann–Whitney U tests were used to examine subgroup differences in perceived usefulness, perceived ease of use, attitude toward using, behavioral intention, and overall usability. The subgroup comparison of learning gain is presented in Table 3.

**Table 3:** Subgroup comparison of gain scores by prior gaming experience.

Outcome	Experienced (*n* = 26), M ± SD	Non-experienced (n = 16), M ± SD	*t*(40)	*p*	Cohen’s *d*
CCKT gain (%)	11.54 ± 14.88	11.56 ± 13.13	−0.01	.996	0.00

As shown in Tables 3 and 4, the two groups did not differ significantly in CCKT gain, *t*(40) = −0.01, *p* = .996, Cohen’s *d* = 0.00. In addition, Mann–Whitney U tests showed no significant subgroup differences in PU (*U* = 196.50, *p* = .765), PEOU (*U* = 178.50, *p* = .444), ATT (*U* = 166.50, *p* = .280), BI (*U* = 164.50, *p* = .257), or SUS total score (*U* = 171.00, *p* = .332).

**Table 4:** Subgroup comparisons of TAM and SUS by prior gaming experience.

Outcome	Experienced (*n* = 26), Mean Rank	Non-experienced (*n* = 16), Mean Rank	*U*	*Z*	*p*
PU	21.06	22.22	196.50	−0.300	.765
PEOU	20.37	23.34	178.50	−0.766	.444
ATT	23.10	18.91	166.50	−1.081	.280
BI	19.83	24.22	164.50	−1.135	.257
SUS	22.92	19.19	171.00	−0.969	.332

Overall, no statistically significant subgroup differences were observed in learning gain, technology acceptance, or overall usability between participants with and without prior gaming experience (all *p* > .05).

### Technical performance and post-exposure discomfort results

The system maintained a stable average frame rate of 62.3 FPS (*SD* = 4.1, 95% CI [60.1, 64.5]) across 15 independent 30-minute continuous runs, and no frame rate drops below 60 FPS were recorded. The mean input-to-action latency was 42 ms (*SD* = 7), indicating responsive real-time interaction during task execution. Participants reported a mean discomfort score of 1.81 (*SD* = 0.61, 95% CI [1.62, 2.00], *N* = 42), while the highest individual mean score was 2.80 on the 5-point scale. No participant discontinued the experiment due to discomfort. Together, these results indicate that the BD-VRG system operated under stable technical conditions with low post-exposure discomfort in the present desktop VR learning context. Complete technical implementation and performance verification results are provided in Table D in S1 File.

### TAM questionnaire

To evaluate students’ acceptance of the BD-VRG system for color composition learning, a TAM questionnaire was administered to 42 participants. Given the moderate sample size (*N* = 42) and the use of composite dimension scores, one-sample t tests against the neutral midpoint of 4.0 were conducted. As shown in Table 5, all four TAM dimensions scored significantly above the criterion, with mean scores ranging from 5.10 to 5.21 and effect sizes ranging from medium to large (Cohen’s *d* = 0.63–0.82), indicating a generally positive level of system acceptance on the 7-point Likert scale. A Wilcoxon signed-rank test yielded the same conclusion. These effect sizes suggest that the positive TAM ratings were meaningful in magnitude beyond statistical significance. The largest effect was observed for PU, whereas BI showed the smallest but still medium-sized effect.

**Table 5:** TAM results against the neutral midpoint (4.0) (*N* = 42).

TAM Dimensions	*M*	*SD*	95% CI	*t* (41)	*p*	Cohen’s *d*
Perceived Usefulness (PU)	5.21	1.49	[4.76, 5.68]	5.28	< .001	0.82
Perceived Ease of Use (PEOU)	5.11	1.57	[4.62, 5.60]	4.58	< .001	0.71
Attitude Toward Using (ATT)	5.19	1.61	[4.69, 5.69]	4.79	< .001	0.74
Behavioral Intention (BI)	5.10	1.76	[4.55, 5.65]	4.06	< .001	0.63

The mean score for the PU dimension was the highest (*M* = 5.21, *SD* = 1.49), indicating that students perceived the system as helpful for learning design and color coordination skills. This was followed by the mean score for the ATT dimension (*M* = 5.19, *SD* = 1.61), reflecting students’ overall positive attitude toward using the VR system to learn color composition. The PEOU score was slightly lower (*M* = 5.11, *SD* = 1.57), and the BI score was the lowest among the four dimensions (*M* = 5.10, *SD* = 1.76), although both remained significantly above the neutral midpoint. This pattern suggests that, although overall acceptance was positive, perceived ease of use and future intention to use could be further strengthened through subsequent system refinement.

To further examine the core TAM mechanism underlying BI, a supplementary bootstrap path analysis was conducted to test the direct and indirect relationships among PEOU, PU, and BI. Because the tested path model was just-identified (df = 0), conventional global model fit indices were not informative and therefore were not reported. Bootstrap confidence intervals were used to evaluate the significance of the direct and indirect effects. As shown in Table 6, PEOU significantly predicted PU (β = 0.451, BC 95% CI [0.144, 0.668], *p* = .008), and PU significantly predicted BI (β = 0.432, BC 95% CI [0.030, 0.782], *p* = .034). In contrast, the direct effect of PEOU on BI was not significant (β = −0.126, BC 95% CI [−0.503, 0.283], *p* = .621). Importantly, the indirect effect of PEOU on BI through PU was significant (β = 0.195, BC 95% CI [0.014, 0.483], *p* = .024), indicating that PU significantly mediated the relationship between PEOU and BI.

**Table 6:** Bootstrap path analysis results for the core TAM pathway.

Path	Effect type	Standardized estimate (β)	BC 95% CI	*p*
PEOU → PU	Direct	0.451	[0.144, 0.668]	0.008
PU → BI	Direct	0.432	[0.030, 0.782]	0.034
PEOU → BI	Direct	−0.126	[-0.503, 0.283]	0.621
PEOU → PU → BI	Indirect	0.195	[0.014, 0.483]	0.024

In addition to internal consistency analysis, an exploratory factor analysis was conducted on the 15 TAM items using principal axis factoring with Promax rotation. The Kaiser–Meyer–Olkin measure of sampling adequacy was 0.625, and Bartlett’s test of sphericity was significant, χ²(105) = 270.692, *p* < .001. Although the KMO value indicated modest sampling adequacy, the significant Bartlett’s test and the theoretically specified TAM structure supported the use of EFA as a preliminary exploratory analysis. A four-factor solution was extracted in line with the theoretical TAM framework, accounting for 56.60% of the total variance. Among the coefficients shown in the pattern matrix, the lowest factor loading was .402 (PU2 on Factor 1), while the highest cross-loading was .434 (PU3 on Factor 1). The ATT, PEOU, and BI items generally loaded on their expected factors, whereas the PU items showed a relatively less distinct pattern. Thus, the four-factor solution provided preliminary support for the four-dimensional TAM framework, although the PU dimension should be interpreted cautiously. The full pattern matrix is provided in Table E in S1 File.

Fig 6 shows the average scores for each TAM item on a 7-point Likert scale, categorized by dimension (PU, PEOU, ATT, BI). Overall, the average scores for the items were closely clustered, ranging from 5.00 to 5.36, indicating generally positive responses across all TAM items. The PU items were slightly higher overall, with PU1 (“Using this VR system improved my ability to learn color matching.”) receiving the highest mean score (*M* = 5.36). Within the PEOU dimension, some variation was also observed, with PEOU3 and PEOU4 showing slightly higher scores than PEOU1 and PEOU2. At the item level, the pattern was broadly consistent with the dimension-level results reported in Table 5. These item-level results complement the dimension-level findings by showing the distribution of responses across individual TAM items.

**Fig 6:**
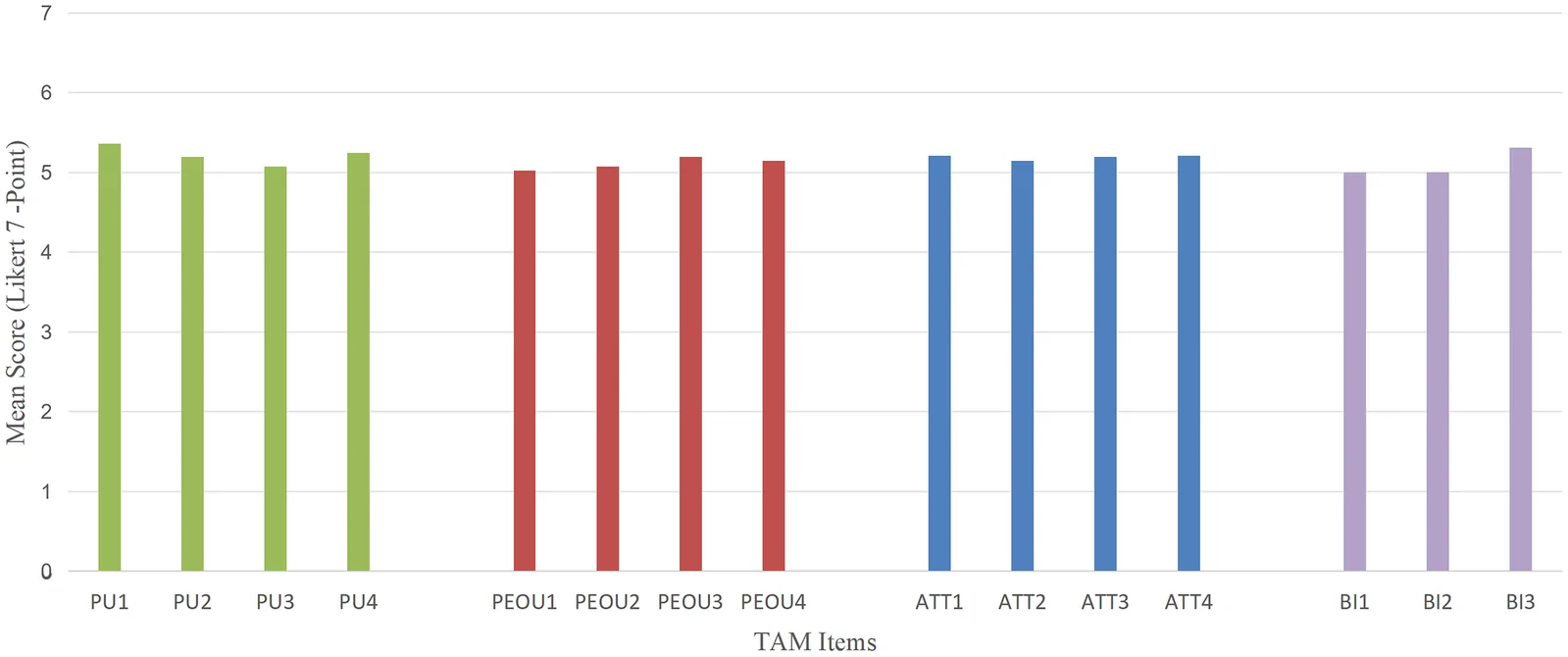
Mean scores of individual TAM items.

Two open-ended questions were included at the end of the TAM questionnaire to collect participants’ feedback on the system’s learning support and unmet needs. These responses were analyzed separately and are reported in the qualitative findings section.

### SUS scale

The SUS scores were calculated based on participants’ responses to ten items, with alternating positive and negative statements. As shown in Table 7, the overall mean SUS score for assessing usability was 75.30 (*SD* = 9.53, 95% CI [72.33, 78.27]) out of 100, exceeding the benchmark score of 68 and indicating that the system achieved a “good” level of usability [49].

**Table 7:** Descriptive statistics of BD-VRG system SUS total score (*N* = 42).

Scale	*M*	*SD*	95% CI
SUS Total	75.30	9.53	[72.33, 78.27]

Item-level responses were generally favorable. Items 3, 1, 7, and 9 received the highest ratings, indicating that participants generally considered the system easy to use, quick to learn, and confidence-enhancing. In particular, Item 3 achieved the highest mean score (*M* = 3.60, *SD* = 0.50), suggesting that ease of use was a notable strength of the system. In contrast, items 10, 4, and 2 had the lowest mean scores, with Item 10 obtaining the lowest rating (*M* = 2.33, *SD* = 0.57). This suggests that some participants perceived a moderate learning curve or occasional need for technical support. Complete item-level descriptive statistics are provided in Table F (S1 File).

Two open-ended questions were included at the end of the SUS scale to collect participants’ feedback on operation difficulties and interface improvement needs. These responses were analyzed separately and are presented in the qualitative findings section.

### Qualitative findings from open-ended feedback

To enrich the interpretation of the quantitative findings, participants’ open-ended responses were analyzed thematically. Six recurring themes were identified, and the major themes, frequencies, percentages, and representative quotations are presented in Table 8.

**Table 8:** Thematic analysis of open-ended responses (*N* = 42).

Theme	Frequency (n)	Percentage (%)	Representative quotation
Real-time feedback was highly useful	25	59.5%	“Seeing the color harmony result immediately helped me know where the problem was.”
The interface and navigation were not intuitive enough	18	42.9%	“The buttons were too small, and I could not find the palette at first.”
Need for beginner-oriented guidance or tutorial	12	28.6%	“It would be better if there were a one-minute tutorial video at the beginning.”
Need for customizable or removable prompts	9	21.4%	“After becoming familiar with it, I would like to turn off those prompt texts.”
Minor lag or responsiveness issues	5	11.9%	“It lagged slightly when I dragged the color bar quickly.”
Increased enjoyment and willingness to practice	21	50.0%	“It was much more interesting than drawing a color wheel on paper.”

The most frequently reported theme was the usefulness of real-time feedback (*n* = 25, 59.5%). Participants generally perceived immediate feedback on color harmony as helpful for identifying mismatched color combinations and making timely adjustments during practice. As one participant stated, “Seeing the color harmony result immediately helped me know where the problem was.” Another prominent positive theme was that the system made learning more interesting and increased willingness to practice (*n* = 21, 50.0%). For example, one participant noted that “It was much more interesting than drawing a color wheel on paper.” These positive themes are broadly consistent with the relatively favorable ratings of perceived usefulness and attitude toward using in the TAM results.

Despite these positive experiences, several usability-related concerns were also evident. Interface and navigation issues were reported by 18 participants (42.9%), indicating that some learners experienced difficulty locating functions or understanding interface elements during initial use. As one participant commented, “The buttons were too small, and I could not find the palette at first.” In addition, 12 participants (28.6%) expressed a need for beginner-oriented guidance or a short tutorial at the beginning of the session. A smaller proportion of participants suggested that prompts should be customizable or removable once they became more familiar with the system (*n* = 9, 21.4%), while minor concerns about system responsiveness were mentioned by five participants (11.9%), especially during rapid color adjustment. Overall, these qualitative findings complement the TAM and SUS results by showing that participants valued the system’s immediate learning support and engaging experience, while also highlighting areas for improvement in navigation clarity, onboarding support, adaptive prompting, and interface responsiveness.

## Discussion

This study developed and evaluated BD-VRG as a constructivist-based desktop VR serious game for color composition learning in basic design education. Participants demonstrated a significant short-term improvement in CCKT performance and reported generally positive technology acceptance and favorable usability. Technical monitoring indicated stable performance and low post-exposure discomfort under the tested conditions. Qualitative feedback further showed that participants valued the system’s real-time feedback and engaging practice, while identifying onboarding and navigation as the main areas requiring improvement.

Participants’ CCKT performance showed significant short-term improvement, with a large within-group effect (*d* = 0.82). This finding aligns with Özgen et al. [17], who identified VR as a promising complement to problem-solving activities in basic design education, and Villada Castillo et al. [9], who reported significant pre–post gains in learning abstract concepts of projectile motion using an immersive VR serious game that supported interactive visualization. From a constructivist perspective, the observed gain is consistent with the emphasis on active exploration, interaction, and meaning construction [37,38]. The findings therefore provide preliminary empirical support for applying constructivist principles to abstract perceptual learning in desktop VR-based design education. BD-VRG may support the learning of abstract color composition concepts through immersive visualization, task-based practice, and immediate feedback. Accordingly, desktop VR environments for design theory courses may benefit from aligning visual exploration, perceptual judgment, and timely scaffolded feedback with explicit learning objectives to promote active meaning construction rather than rote memorization.

The combined TAM, SUS, and qualitative findings further suggest that students primarily valued the system as a learning support tool rather than merely as a novel digital environment. The medium-to-large TAM effects indicate that students’ positive acceptance was meaningful in magnitude, the strongest effect was observed for perceived usefulness, followed closely by attitude toward using. From a practical perspective, this pattern suggests that students recognized the learning value of BD-VRG for color composition practice and responded positively to the learning experience. This emphasis on perceived learning value is broadly consistent with Wang et al. [4], who found that perceived usefulness significantly predicted attitude toward using a mobile color-mixing game. By presenting abstract color relationships more intuitively, the immersive features of BD-VRG may have supported task-related learning and perceived usefulness, while its gamified elements may have contributed to positive attitudes. These interpretations are broadly consistent with evidence that learners may value the learning support provided by educational VR games even when some operational complexity remains [52] and that game elements in VR educational environments can stimulate motivation and interest [27]. The qualitative findings reinforce this interpretation. In particular, the most frequently reported theme was the usefulness of real-time feedback, and many participants also reported increased enjoyment and willingness to practice.

By contrast, perceived ease of use and behavioral intention were relatively lower, although both remained significantly above the neutral midpoint. As behavioral intention had the lowest mean score and the smallest effect size among the four TAM dimensions, the findings suggest that positive evaluations of the system were not accompanied by equally strong future use intention. This pattern may relate to ease of use, as suggested by the item-level SUS results and qualitative findings. Higher scores on SUS Items 3 and 7 suggest that many students found the system easy to use and learn, while the favorable score for Item 1 is also consistent with the qualitative theme of increased enjoyment and willingness to practice. In contrast, the relatively lower scores on SUS Items 10, 4, and 2, despite remaining above the midpoint, indicate an initial learning burden, a need for support, or perceived complexity. These findings align with qualitative comments about navigation problems, difficulty locating the palette, small buttons, and the need for beginner-oriented guidance. Therefore, these findings have practical implications for system refinement: future iterations should maintain the system’s perceived learning value while improving interaction flow and navigation clarity, and should provide more flexible interface guidance as users become more familiar with the system.

The supplementary path analysis further showed that perceived ease of use influenced behavioral intention indirectly through perceived usefulness, consistent with the core TAM mechanism [43] and the PU-mediated pathway reported by Al-Adwan et al. [53], who likewise found no direct PEOU–BI effect. This pattern contrasts with Issayeva et al. [54], who reported significant direct effects of both perceived ease of use and perceived usefulness on higher education students’ intention to adopt VR. One possible explanation is that, after using a specific educational system, students may place greater emphasis on its learning value than on operational convenience alone. Accordingly, in the post-use evaluation of BD-VRG, ease of use may have functioned primarily as a value-enabling condition, contributing to future use intention by enhancing the system’s perceived usefulness for color composition learning.

Another noteworthy finding is that prior gaming experience was not associated with statistically significant differences in learning gain, technology acceptance, or perceived usability. Within this sample, prior gaming experience therefore did not appear to be a prerequisite for achieving learning gains or positive system evaluations. This finding suggests the potential for broader classroom implementation of desktop VR in basic design education and aligns with current educational VR trends emphasizing accessibility and classroom integration [55]. Such accessibility is particularly relevant for classrooms involving learners with diverse digital backgrounds [56]. In addition, the system showed stable technical performance, low latency, and low post-exposure discomfort, which are important prerequisites for sustained instructional use in art and design learning [47]. Although a small number of participants mentioned minor lag or responsiveness issues in the qualitative feedback, these comments appear to reflect localized interaction experiences rather than broader technical instability.

Overall, these findings indicate that BD-VRG shows promise as a low-cost and classroom-deployable learning tool for basic design education, although further refinement is needed in interface clarity, onboarding, and interaction smoothness. Continued improvement in these areas may help enhance the overall user experience and support more effective routine classroom use.

## Conclusions

This study developed and evaluated BD-VRG, a constructivist-based desktop VR serious game for color composition learning in basic design education. The findings indicate that the system shows promise as a low-cost, classroom-deployable learning tool, with positive results in short-term performance, learner acceptance, usability, and technical feasibility.

Beyond these immediate findings, the study contributes to the field in two main ways. First, it provides preliminary empirical evidence on the use of desktop VR serious games for abstract learning tasks in basic design education. The focus on desktop VR was intentional, as it offers lower cost, easier deployment, and greater classroom practicality than head-mounted alternatives. The observed short-term CCKT improvement (*d* = 0.82) suggests that high-fidelity embodied immersion may not be the only viable approach for supporting abstract color composition learning. Second, the combined quantitative and qualitative findings suggest that the perceived educational value of the system may be associated not only with the desktop VR environment but also with its constructivist serious game design, particularly its use of task-based progression, immediate feedback, and repeated interactive practice. Taken together, these findings highlight the potential of desktop VR serious games as accessible and potentially scalable tools for supporting interactive learning in basic design education, thereby potentially helping to address an important accessibility gap in this area.

Nevertheless, several limitations should be acknowledged. This exploratory single-group pre–post study was intended to examine feasibility and short-term performance changes rather than establish causal evidence of instructional effectiveness. Without a control condition, the significant pre–post gain and large within-group effect size cannot be attributed solely to BD-VRG, as testing and familiarity effects cannot be excluded. The modest sample of first-year students from a single Chinese university (*N* = 42) limits the precision of the estimates and the generalizability of the findings; therefore, the associated estimates and confidence intervals should be interpreted cautiously, and the findings require replication in larger and more diverse samples. Given these constraints, the reliability estimates for the CCKT, although acceptable for both forms, should be interpreted as preliminary, and strict empirical equivalence between the two CCKT forms should be further examined in future studies using independent counterbalanced administration. In addition, the EFA results for the adapted TAM questionnaire should be interpreted cautiously in light of the exploratory nature of the analysis and the modest KMO value, and the factor structure requires further examination. Future research should also use randomized controlled designs and longer intervention periods to assess causal and longer-term effects. Despite these limitations, the study provides a practical foundation for subsequent research by documenting the development, classroom implementation, usability, learner acceptance, technical performance, and refinement needs of a low-cost desktop VR serious game.

## Supporting information

S1 File Appendices A-D and Tables A-F. (DOCX)

S2 File CCKT pre-test and post-test data. (XLSX)

S3 File TAM questionnaire data. (XLSX)

S4 File SUS data. (XLSX)

S5 File SSQ-5 data. (XLSX)
