# Supplementary material for: Development and evaluation of a desktop VR serious game for color composition learning in basic design education
Source: PLoS One. 2026 Sep 30;21(9):e0359618. doi: 10.1371/journal.pone.0359618 (PMC13626362; doi:10.1371/journal.pone.0359618)
Supplement: Supplementary file 1 [file pone.0359618.s001.docx]

# **Appendix A**

**Needs-Assessment Raw Themes (N = 25)**

**1. Data Source and Participants**

A needs assessment was conducted during the analysis stage of the ADDIE process to identify real classroom challenges and learner expectations relevant to color harmony learning in basic design education. Data were collected from two sources: (1) semi-structured interviews with lecturers (N = 2) of a basic design course at a university in Shandong Province, and (2) an open-ended questionnaire administered to first-year undergraduate art and design students (N = 25). Participation was voluntary. No personally identifying information was collected, and responses were anonymized prior to analysis.

**2. Open-Ended Questionnaire Prompts (Student Needs Assessment)**

Q1. What aspects of color composition do you find most difficult to understand in the basic design course? Please describe briefly.

Q2. What kinds of learning support do you expect to help you better understand and apply color harmony rules (e.g., examples, practice, feedback)?

Q3. What learning activities or formats would increase your willingness to engage with color theory and practice?

Q4. What features would you expect from a digital/interactive learning tool for color learning?

**3. Coding and Theme Derivation**

Responses were summarized using an inductive approach to extract recurring needs and learning barriers relevant to basic color knowledge, color harmony perception, and color composition practice. The analysis followed these steps:

1. **Data preparation:**

All student responses were exported and anonymized.

2. **Initial coding:**

The researcher conducted open coding line-by-line to label meaningful units related to learning difficulties, desired support, and expected learning experiences.

3. **Theme generation:**

Similar codes were grouped into broader themes and sub-themes through iterative review.

4. **Frequency calculation:**

Each response could contribute to more than one theme. Theme frequency was calculated as the number of participants who mentioned a given theme at least once (n), divided by N = 25 (%).

5. **Auditability:**

The final raw themes and frequencies are reported in Table A1.

**4. Needs-Assessment Themes and Frequencies**

| Table A1. Themes derived from student open-ended responses (N = 25) | | | |
| --- | --- | --- | --- |
| Theme | Operational definition | Frequency (n) | Percentage (%) |
| T1. Color theory is abstract/difficult to understand | Mentions abstractness, hard to visualize, hard to apply color rules | 20 | 80% |
| T1.1 Confusion about hue/purity/brightness | Mentions difficulty distinguishing or relating basic attributes | 16 | 64% |
| T1.2 Difficulty applying harmony rules | Mentions complementary /analogous/contrast rules are confusing | 18 | 72% |
| T2. Traditional learning lacks interactivity / relies on static tools | Mentions PPT/textbook-only, lack of hands-on practice/experiments | 24 | 96% |
| T2.1 Need trial-and-error practice | Mentions wanting to try combinations and see outcomes | 11 | 44% |
| T2.2 Need immediate/explicit feedback | Mentions needing score, hints, explanations, review | 9 | 36% |
| T3. Low motivation/engagement | Mentions boredom, low willingness, hard to persist | 17 | 68% |
| T3.1 Preference for gamified tasks/rewards | Mentions levels, missions, scoring, badges, rewards, challenge to increase motivation | 11 | 44% |
| T3.2 Desire for authentic examples | Mentions product/poster cases, realistic examples | 22 | 88% |
| T4. Desired features for a digital learning tool | Mentions tool features or preferred interactive modality for learning color composition | 19 | 76% |
| T4.1 Desire richer color resources | Mentions more swatches /palettes/styles/examples | 17 | 68% |
| T4.2 Preference for immersive/VR/3D interactive learning modality | Mentions VR, 3D virtual environments, immersive scenes, or interactive simulation | 12 | 48% |

Note. Frequency (n) indicates the number of participants who mentioned the theme at least once. Participants may be coded into multiple themes; therefore, totals may exceed N = 25 and percentages do not necessarily sum to 100%. Parent themes are umbrella categories used to organize subthemes; subtheme frequencies should not be summed.

**5. Requirement extraction (R1–R6)**

Based on the needs-assessment themes summarized in Table A1, the following design requirements were extracted to guide the BD-VRG development:

**R1 Concept visualization & concreteness**: Provide interactive visualization to reduce the abstractness of color theory (supports T1/T1.1/T1.2).

**R2 Hands-on practice**: Support trial-and-error practice activities to reinforce learning through repeated experimentation (supports T2/T2.1).

**R3 Immediate and explicit feedback**: Provide instant, explicit feedback (e.g., correctness prompts, scores, review) to support error correction and reinforcement (supports T2.2).

**R4 Engagement support**: Incorporate gamified challenges and reward-related mechanisms to increase motivation and persistence (supports T3/T3.1).

**R5 Authentic learning support**: Provide authentic examples and scenario cues to connect color theory with real design practice (supports T1–T3 broadly).

**R6** **Accessible immersive modality**: Support immersive/3D interaction in a deployable, low-barrier desktop VR format suitable for classroom use (supports T4/T4.1).

# **Appendix B**

**Expert Review Form (Two Rounds)**

**1. Expert profile (completed once per expert)**

**Expert ID:** E1 / E2

**Expertise:** □ Basic design education □ Color theory □ Educational technology □ HCI/UX □ Other: ____

Years of relevant experience: ____

Role: □ Lecturer □ Researcher □ Practitioner □ Other: ____

**2. Review instructions**

**Decision label:**

A = Accept (no revision needed)

R = Revise (minor/major revision needed)

X = Reject (major redesign required)

**Coding rule:** *κ* is calculated on the decision label (A/R/X) across items.

**3. Evaluation Form:** Same items for both rounds

| **Evaluation Items** | | **Decision label (A/R/X)** | **Comments (required for R/X)** |
| --- | --- | --- | --- |
| 1 | Alignment between instructional objectives and learning tasks (color concepts and harmony rules are operationalized appropriately) |  |  |
| 2 | Pedagogical coherence of the learning flow and appropriateness of scaffolding/difficulty progression |  |  |
| 3 | Accuracy and appropriateness of terminology and instructional language for first-year art/design students |  |  |
| 4 | Interface information hierarchy is clear (layout, grouping, and readability support task focus) |  |  |
| 5 | Interaction logic is consistent with the intended workflow (button placement/labeling reduces ambiguity) |  |  |
| 6 | Feedback design is clear and actionable (e.g., completion confirmation, error prompts, next-step guidance) |  |  |
| 7 | Gamification/reward design is motivating without distracting from learning objectives |  |  |
| 8 | System stability and technical robustness are adequate for planned classroom operation (e.g., no freezing/crashing in typical use) |  |  |
| 9 | Desktop VR configuration is feasible for classroom deployment (setup complexity/time, equipment availability, user operation) |  |  |
| 10 | Overall feasibility, completeness, and internal consistency of the BD-VRG design |  |  |

Round summary:

Major issues identified: ____

Key revisions implemented: ____

Overall decision for this round: □ Proceed □ Minor revision □ Major revision

**4. Inter-rater agreement (κ) reporting**

Inter-rater agreement between the two experts was assessed using Cohen’s κ based on the decision labels (A/R/X) assigned to each of the 10 evaluation items. Round 1 *κ* = 0.86 and Round 2 *κ* = 0.83.

# **Appendix C**

**Iteration log of BD-VRG development (v0.1-v1.0)**

**Purpose：**

This appendix documents the iterative refinement of BD-VRG from the first functional draft to the final prototype, providing a traceable audit trail from identified needs (Appendix A), expert review (Appendix B), and internal testing to implementation updates described in this study.

**Table 1. Version-by-version iteration log**

| **Version** | **Date** | **Issue identified** | **Revision action** | **Evidence source** | **Status** |
| --- | --- | --- | --- | --- | --- |
| v0.1 | 2025-04 (Weeks 1–2) | Need a low-barrier classroom-deployable baseline | Implemented desktop VR baseline and basic scene layout to ensure low-barrier classroom deployability | Appendix A (A5:R6) | Completed |
| v0.3 | 2025-04–2025-05 (Weeks 3–4) | Learning content needed to be structured into runnable modules | Implemented theoretical module content delivery (color wheel / emotional expression / visual perception) and module access flow | Appendix A (A5:R1) | Completed |
| v0.4 | 2025-05 (Week 5) | Tasks required operationalization for practice and assessment | Implemented game panel activation and task scripts enabling color selection/  modification /composition | Appendix A (A5:R2) | Completed |
| v0.5 | 2025-05 (Week 6) | Expert review required clearer task feedback and instructional process | Integrated task practice with matching/mismatching feedback prompts and score output | Appendix B (Item 6) | Completed |
| v0.6 | 2025-05–2025-06 (Week 7) | Expert review required learning-aligned gamification | Added progress wall + badges and repeat-challenge mechanism tied to task performance | Appendix B (Item 7) | Completed |
| v0.7 | 2025-06 (Week 8) | Expert review required a more reasonable difficulty progression | Adjusted task/level sequence and calibrated hint/scaffolding intensity across modules | Appendix B (Item 2) | Completed |
| v1.0 | 2025-06–2025-07 (Weeks 9–10) | Final prototype for evaluation | Packaged as a Windows executable and instrumented performance logging (FPS/interaction latency); SSQ-5 prepared. | Table D | Completed |

# **Appendix D**

**Test blueprint and representative items from the two parallel forms of the Color Composition Knowledge Test (CCKT)**

This appendix presents the test blueprint and representative items from the two parallel forms of the CCKT. The items were developed based on the course learning objectives, the instructional content of the BD-VRG modules, and established color-composition teaching materials. A common test blueprint was used to ensure comparable coverage of the target knowledge domains across the pre-test and post-test forms, as shown in Table 1.

**Table 1. Test blueprint for the two parallel forms of the CCKT**

| **Core theme** | **Target knowledge domain** | **Intended learning objective** | **Pre-test items** | **Post-test items** | **No. of items** |
| --- | --- | --- | --- | --- | --- |
| Foundational knowledge of color | Basic color properties, color matching, and color relationships | Identify and apply basic color attributes, matching principles, and common color relationships | Pre_Q1–Pre_Q10 | Post_Q1–Post_Q10 | 10 |
| Emotional expression of color | Emotional associations of color | Match color combinations with intended emotional meanings | Pre_Q11–Pre_Q15 | Post_Q11–Post_Q15 | 5 |
| Visual perception of color composition | Overall harmony judgment | Evaluate harmony, proportion, brightness balance, and visual coordination | Pre_Q16–Pre_Q20 | Post_Q16–Post_Q20 | 5 |

**Note. Item numbers refer to the corresponding items in the pre-test and post-test forms.**

The representative paired items below illustrate the scope, format, and intended difficulty of the test. Each displayed pair assesses the same target concept. These items are not intended to represent the full item pool. Each item requires participants to select the most appropriate answer from four response options.

**Pair 1. Complementary color relationship**

**Correspondence:** Pre-test Item 1 ↔ Post-test Item 1

**Target concept:** Identification of complementary color relationships

**Pre-test Item 1**

A student wants to create a strong contrast by selecting two colors located opposite each other on the color wheel. Which pair is the most appropriate?

A. Red and orange

B. Blue and green

C. Yellow and purple

D. Yellow and yellow green

**Correct answer: C**

**Post-test Item 1**

In a package design task, which color pair would be most suitable for producing a high-contrast effect based on a complementary relationship?

A. Blue and blue green

B. Red and green

C. Yellow and orange

D. Purple and red

**Correct answer: B**

**Pair 2. Analogous color relationship**

**Correspondence:** Pre-test Item 2 ↔ Post-test Item 2

**Target concept:** Identification of analogous color schemes

**Pre-test Item 2**

Which of the following sets of colors is most appropriate for creating a smooth and coordinated analogous scheme?

A. Blue, blue-green, and green

B. Red and green

C. Yellow and purple

D. Orange and blue

**Correct answer: A**

**Post-test Item 2**

A designer wants to produce a visually unified composition using neighboring hues on the color wheel. Which group of colors best fits this purpose?

A. Red, red-orange, and orange

B. Blue and orange

C. Green and red

D. Yellow and purple

**Correct answer:** **A**

**Pair 3. Warm–cool contrast**

**Correspondence:** Pre-test Item 3 ↔ Post-test Item 3

**Target concept:** Recognition of warm–cool contrast

**Pre-test Item 3**

Which color combination would most clearly express a warm–cool contrast in a composition?

A. Yellow and orange

B. Blue and green

C. Orange and blue

D. Red and orange red

**Correct answer: C**

**Post-test Item 3**

Which pair of colors would best create a warm–cool contrast in a design?

A. Purple and blue

B. Red and orange

C. Yellow and red

D. Blue and orange red

**Correct answer: D**

**Pair 4. Emotional association of color**

**Correspondence:** Pre-test Item 11 ↔ Post-test Item 11

**Target concept:** Matching color combinations to emotional tone

**Pre-test Item 11**

If a design theme aims to communicate a calm and peaceful mood, which color combination is the most appropriate?

A. Bright red and orange

B. Blue and light green

C. Yellow and red

D. Black and dark red

**Correct answer: B**

**Post-test Item 11**

If a design is intended to create a quiet and soothing visual mood, which color combination is the most appropriate?

A. Soft blue and blue green

B. Bright yellow and orange

C. Red and black

D. Purple and red

**Correct answer: A**

**Pair 5. Overall harmony judgment**

**Correspondence:** Pre-test Item 16 ↔ Post-test Item 16

**Target concept:** Judgment of overall harmony in color composition

**Pre-test Item 16**

Which of the following design choices is most likely to produce a harmonious visual effect?

A. Combining highly saturated complementary colors in equal proportions without adjustment

B. Using related hues with controlled brightness differences and balanced proportions

C. Arranging several unrelated strong colors without a clear visual hierarchy

D. Mixing multiple intense colors only to increase visual stimulation

**Correct answer: B**

**Post-test Item 16**

Which of the following color arrangements is most likely to create overall harmony in a composition?

A. Repeating several unrelated saturated colors evenly across the page

B. Using only the brightest available colors to strengthen visual impact

C. Selecting related hues and adjusting proportion and brightness balance carefully

D. Mixing warm and cool colors randomly to increase complexity

**Correct answer: C**

# **Table A. Requirement-to-design mapping for BD-VRG.**

| **Requirement** | **Design decision** | **Where reported** |
| --- | --- | --- |
| R1. Concept visualization & concreteness | VR simulation for color learning; immersive and risk-free space to master abstract color theory through interactive learning | Design (VR simulation; immersive learning).  (master abstract concepts via VR) |
| R2. Hands-on practice | Module-driven tasks as practical training following theoretical learning. | Course content design (immediate practice after concepts) |
| R3. Immediate and explicit feedback | Task scripts recognize matching and provide feedback prompts and scoring | Design (instant feedback)  Development (feedback & scores; matching prompts) |
| R4. Engagement support | Reward system and progress wall displaying total score, completed tasks, earned badges; repeat challenges to improve scores | Development (progress wall/badges/repeat challenges) |
| R5. Authentic learning support | Three themes: foundational knowledge, emotional expression, visual perception | Course content design |
| R6. Accessible immersive modality | Desktop VR setup without HMD; Windows executable for desktop VR operation | Experimental Environment and Equipment |

# **Table B. Summary of expert-review revisions across two rounds**

|  | **Expert suggestion** | **Design update implemented** |
| --- | --- | --- |
| 1 | Make the “explain–practice–feedback–application” flow operational. | Clarified the operational learning flow by linking the core color theory via interactive display to task execution through activating the game practice, followed by feedback and application. |
| 2 | Keep terminology consistent; avoid vague prompts. | Standardized instructional wording across modules, such as color wheel / emotional expression / visual perception descriptions. |
| 3 | Ensure clear task feedback | Add score pop-up/hint text; key actions were made explicit through consistent feedback cues |
| 4 | Rewards should reflect learning performance, not random completion | Clarified reward logic in the design specification. The progress wall and virtual interface display the total score, completed tasks, and badges, and learners can repeat challenges to improve scores |
| 5 | Specify to prevent error-prone operations | Users can exit via the escape button, while a pop-up confirmation prompt prevents unintentional exits/shutdowns |
| 6 | Ensure a reasonable difficulty gradient | Adjust level order and hint intensity to form a coherent difficulty progression from basic concept discrimination to higher-level tasks. |

# **Table C. Item-level descriptive statistics for the pre-test and post-test forms of the Color Composition Knowledge Test (CCKT).**

Difficulty values are reported as the proportion of correct responses, and discrimination is reported as corrected item–total correlations. Item-level descriptive analyses were conducted separately for the pre-test and post-test forms using response data from the formal study sample (N = 42).

**Table C1. Pre-test form**

| **Item** | **Difficulty (p)** | **Corrected item–total correlation** |
| --- | --- | --- |
| Pre_Q1 | 0.548 | 0.076 |
| Pre_Q2 | 0.548 | 0.230 |
| Pre_Q3 | 0.619 | 0.296 |
| Pre_Q4 | 0.452 | 0.258 |
| Pre_Q5 | 0.452 | 0.476 |
| Pre_Q6 | 0.500 | 0.375 |
| Pre_Q7 | 0.405 | 0.239 |
| Pre_Q8 | 0.619 | 0.310 |
| Pre_Q9 | 0.452 | 0.311 |
| Pre_Q10 | 0.357 | 0.377 |
| Pre_Q11 | 0.429 | 0.383 |
| Pre_Q12 | 0.429 | 0.301 |
| Pre_Q13 | 0.405 | 0.334 |
| Pre_Q14 | 0.500 | 0.164 |
| Pre_Q15 | 0.595 | 0.359 |
| Pre_Q16 | 0.500 | 0.203 |
| Pre_Q17 | 0.429 | 0.079 |
| Pre_Q18 | 0.643 | 0.317 |
| Pre_Q19 | 0.524 | 0.282 |
| Pre_Q20 | 0.619 | 0.269 |

**Table C2. Post-test form**

| **Item** | **Difficulty (p)** | **Corrected item–total correlation** |
| --- | --- | --- |
| Post_Q1 | 0.643 | 0.195 |
| Post_Q2 | 0.690 | 0.325 |
| Post_Q3 | 0.690 | 0.284 |
| Post_Q4 | 0.643 | 0.195 |
| Post_Q5 | 0.619 | 0.542 |
| Post_Q6 | 0.690 | 0.229 |
| Post_Q7 | 0.571 | 0.309 |
| Post_Q8 | 0.643 | 0.092 |
| Post_Q9 | 0.667 | 0.264 |
| Post_Q10 | 0.595 | 0.375 |
| Post_Q11 | 0.548 | 0.388 |
| Post_Q12 | 0.643 | 0.234 |
| Post_Q13 | 0.476 | 0.506 |
| Post_Q14 | 0.524 | 0.111 |
| Post_Q15 | 0.690 | 0.082 |
| Post_Q16 | 0.619 | 0.542 |
| Post_Q17 | 0.524 | 0.470 |
| Post_Q18 | 0.500 | 0.381 |
| Post_Q19 | 0.690 | 0.311 |
| Post_Q20 | 0.667 | 0.457 |

# **Table D. Technical implementation and performance verification of the BD-VRG**

| **Item** | **Specification / Measured Result** | **Notes** |
| --- | --- | --- |
| Game engine | Unity 2022.3.15f1 LTS (Built-in Render Pipeline) | Ensured stable operation on standard classroom computer-lab PCs |
| Display device | 24-inch Full HD monitor (1920 × 1080, 60 Hz) | Standard university computer-lab configuration; no stereoscopic display |
| Input device | Standard USB keyboard (WASD movement) + mouse (view control / clicking / dragging) | No tracking devices required |
| Average frame rate (FPS) | 62.3 ± 4.1 (95% CI [60.1, 64.5], *N* = 15 continuous 30-minute runs) | Measured using Unity Profiler; all test sessions remained above 60 FPS with no frame drops |
| Input-to-action latency | 42 ± 7 ms | Time from mouse click to object response; met real-time interaction requirements |
| Brief post-exposure discomfort measure (SSQ-5) | Mean = 1.81 (*SD* = 0.61, 95% CI [1.62, 2.00], *N* = 42) | Highest individual mean score was 2.8/5; no participant withdrew due to discomfort; meeting the low-risk expectations for desktop VR. |
| Test hardware configuration | Intel i7-9700, 16 GB RAM, GTX 1660 | Uniform laboratory setup; all 42 participants used the same hardware |
| Build output | Standalone executable file (.exe, 182 MB) | No Unity installation or additional plugins required |

# **Table E. Pattern matrix of the adapted TAM questionnaire**

| **Item** | **Factor 1** | **Factor 2** | **Factor 3** | **Factor 4** | **Theoretical construct** |
| --- | --- | --- | --- | --- | --- |
| BI2 | .967 |  |  |  | BI |
| BI3 | .711 |  |  |  | BI |
| BI1 | .641 |  |  |  | BI |
| PU2 | .402 |  |  |  | PU |
| PEOU2 |  | .977 |  |  | PEOU |
| PEOU1 |  | .658 |  |  | PEOU |
| PEOU4 |  | .621 |  |  | PEOU |
| PEOU3 |  | .463 |  |  | PEOU |
| ATT1 |  |  | .781 |  | ATT |
| ATT2 |  |  | .780 |  | ATT |
| ATT3 |  |  | .764 |  | ATT |
| ATT4 |  |  | .622 |  | ATT |
| PU1 |  |  |  | .783 | PU |
| PU4 |  |  |  | .664 | PU |
| PU3 | .434 |  |  | .491 | PU |

# **Table F. Descriptive statistics of each SUS item**

| **No.** | **Items** | ***M*** | ***SD*** |
| --- | --- | --- | --- |
| 1 | I think that I would like to use this system frequently. | 3.48 | 0.55 |
| 2 | I found the system unnecessarily complex. | 2.67 | 0.57 |
| 3 | I thought the system was easy to use. | 3.60 | 0.50 |
| 4 | I think that I would need the support of a technical person to be able to use this system. | 2.40 | 0.63 |
| 5 | I found the various functions in this system were well integrated. | 3.31 | 0.52 |
| 6 | I thought there was too much inconsistency in this system. | 2.76 | 0.53 |
| 7 | I would imagine that most people would learn to use this system very quickly. | 3.43 | 0.59 |
| 8 | I found the system very cumbersome to use. | 2.74 | 0.59 |
| 9 | I felt very confident using the system. | 3.40 | 0.54 |
| 10 | I needed to learn a lot of things before I could get going with this system. | 2.33 | 0.57 |
